# Prevalence and degree of orotracheal intubation‐related tracheal lesions in horses

**DOI:** 10.1111/evj.14487

**Published:** 2025-02-18

**Authors:** Aiden Parente, Florian Geburek, Sabine Kästner, Charlotte Iversen, Klaus Hopster

**Affiliations:** ^1^ New Bolton Center University of Pennsylvania Kennett Square Pennsylvania USA; ^2^ Clinic for Horses University of Veterinary Medicine Hannover, Foundation Hannover Germany; ^3^ Department of Veterinary Clinical Studies University of Copenhagen Copenhagen Denmark

**Keywords:** anaesthesia, endotracheal tube, horse, intubation, trachea

## Abstract

**Background:**

Orotracheal intubation is commonly performed for inhalation anaesthesia in horses to ensure safe and reliable administration of volatile anaesthetics and to secure the airways. In human and equine medicine, the occurrence of intubation‐associated complications has been described, which can range from mild mucosal irritation to severe necrosis. However, there are only sparse descriptions of mucosal alterations and the course of healing after elective surgery in horses.

**Objective:**

To investigate the prevalence and severity of tracheal lesions following endotracheal intubation associated with general anaesthesia in equine patients.

**Study Design:**

Prospective, clinical study.

**Methods:**

Forty adult Warmblood horses, weighing 400–600 kg, presented for elective surgery under general anaesthesia were included. For orotracheal intubation, a silicone tube was used and inflated until a cuff pressure of 40 cmH_2_O was confirmed. In all horses, endoscopic examination of the trachea with video documentation was performed prior to intubation and immediately after extubation, as well as 1, 3, 5 and 7 days after general anaesthesia. The type (redness, secretion, bleeding, erosions) and degree (0 = non‐existent to 4 = severe) of corresponding lesions were assessed and scored. The occurrences of clinical signs were recorded. Statistical evaluation was performed using Friedman's test and Pearson correlation.

**Results:**

The median score immediately after recovery from anaesthesia was 6 (2–12) and increased significantly to 11 (4–15) after 1 day (*p* = 0.021). At day 7, the median score was 0 (0–2) with only four horses showing signs of mild tracheitis, and clinical signs were not observed throughout this time period. The duration of intubation correlated with the degree of tracheal damage (*r* ^2^ = 0.67, *p* < 0.001).

**Main Limitations:**

Clinical, non‐randomised study without a control group.

**Conclusion:**

Although orotracheal intubation was accompanied by focal inflammation of the trachea, in the present study no horses showed clinical signs and lesions healed within a week.

## 1 INTRODUCTION

Orotracheal intubation is a common procedure in equine anaesthesia, allowing for the administration of inhalant anaesthetics and ensuring a patent airway. Intubation, while generally safe when performed correctly, carries several potential risks and complications.

Tracheal ischaemic lesions are a common complication of intubation and mechanical ventilation in human patients.^1^ Arterial hypotension,^2^ overinflation of the endotracheal tube cuff,^3^, ^4^, ^5^, ^6^ hypoxemia,^7^ but also continuous aspiration of subglottic secretions^8^ have been identified as risk factors for tracheal lesions in animal and human models. More severe complications such as tracheal stenosis,^9^, ^10^, ^11^, ^12^ and ruptures^13^, ^14^ have been observed in humans resulting from the ischaemic lesions and weakening of the tracheal wall.

The severity of ischaemic lesions of the trachea and the subsequent complications have decreased with the succession of low‐volume, high‐pressure cuffs by high‐volume, low‐pressure cuffs in horses.^1^ Still, post‐intubation tracheal lesions remain a risk, although not well documented due to the lack of clinical signs presented. To date, few recent studies,^15^, ^16^, ^17^ describe the ubiquitous nature of tracheal ischaemic lesions in healthy horses. Though prior studies offer a broad perspective on the prevalences and prognosis of these ischaemic lesions,^15^, ^16^, ^17^, ^18^ predictive factors have yet to be isolated and directly studied. By considering potential confounding variables, such as position and mechanical ventilation, this study aims to evaluate the correlation between age, lesion severity and recovery. Based on published studies in other species, we hypothesised that the prevalence and severity of subclinical tracheal lesions following intubation would correlate with the duration of intubation and the age of the patient.

## 2 MATERIALS AND METHODS

### 2.1 Study population

All animals were presented to the Equine Hospital, University of Veterinary Medicine Hannover, Foundation, for elective surgery under general anaesthesia. For inclusion, horses had to be evaluated ASA status I or II, between 3 and 18 years old, with a bodyweight of 400–600 kg, and have been hospitalised for a minimum of 7 days. Systemic health was determined by assessing history, physical exam, and laboratory diagnostics including complete blood count (CBC) and plasma biochemical profile where indicated.

### 2.2 Study design and data collection

Prior to general anaesthesia (0) as well as immediately after recovery (R) and 1, 3, 5 and 7 days after general anaesthesia, videoendoscopy of the trachea was performed with sedation. The videoendoscope was passed through the ventral meatus of the nasal passages, down the trachea to the carina and the images were recorded while moving the videoendoscope orally. Screenshots of the endoscopy included the 15 cm segment of the trachea where measurements indicated the location of the distal end of the endotracheal tube as well as the cuff of the endotracheal tube. These pictures were then uploaded anonymised into a PowerPoint file in randomised order for offline analysis. All pictures were analysed by three different evaluators (ECVAA boarded anaesthetist, ECEIM boarded internist, ECVS boarded surgeon) scoring the degree of redness, haemorrhage, secretion and build‐up, mucosal erosion and mucous membrane replacement (Table 1).

**TABLE 1:** Score chart for degree of trachea irritation.

Score	Degree of visible redness	Degree of visible build‐up	Secretion: Amount and character	Haemorrhage	Degree of mucal erosions	Degree of mucous membrane replacement
0	0%–20%	0%–20%	0%–20%, mainly serous	0%–20%	0%–10%	0%–10%
1	20%–40%	20%–40%	20%–40%, serous/mucous	20%–40%	10%–30%	10%–20%
2	40%–60%	40%–60%	40%–60%, serous/mucous	40%–60%	30%–60%	20%–30%
3	60%–80%	60%–80%	60%–80%, mainly mucous	60%–80%	60%–80%	30%–50%
4	80%–100%	80%–100%	80%–100%, mainly mucous	80%–100%	80%–100%	>50%

*Note*: Total score as sum of the six individual scores: 0, no damage; 1–8, mildly damaged; 9–13, moderately damaged; 14–19, severely damaged; 20–24, highly severely damaged.

*Source*: Modified Ching et al. and Homi et al.^19^, ^20^

### 2.3 Anaesthesia protocol and tracheal intubation

Prior to anaesthesia, a complete physical exam and a CBC were performed for a baseline health status assessment. Horses received systemic antimicrobial agents and systemic nonsteroidal anti‐inflammatories (NSAIDs) based on the procedure performed. Sedation [0.6–1 mg/kg xylazine (Xylavet®, Vetoquinol GmbH) IV], anaesthesia induction [0.05 mg/kg diazepam (DiazepamAbZ® 10 mg, AbZ Pharma GmbH) and 2.2 mg/kg ketamine (Narketan®, Vetoquinol GmbH) IV] and maintenance of anaesthesia with isoflurane (Isofluran® CP, CP‐Pharma) in oxygen were identical in all horses. Lactated Ringer's solution was administered at a rate of 5 mL/kg/h, and dobutamine (Dobutamin‐ratiopharm® 250 mg, ratiopharm GmbH) was given to effect to maintain a mean arterial blood pressure (MAP) above 60 mmHg during anaesthesia. Arterial blood pressure, heart rate, respiratory rate, arterial saturation via pulse oximetry, expired CO_2_ concentration via capnography, inspired oxygen concentration and expiratory isoflurane concentration were monitored and recorded continuously with an anaesthetic multi‐parameter monitor (Cardiocap/5, Datex‐Ohmeda GmbH).

Following the induction of anaesthesia, horses were orotracheally intubated in lateral recumbency using a blind technique. The head and neck were extended in a straight line and the mouth was opened using a mouth gag. A lubricated high volume, low pressure Cook silicone endotracheal tube was inserted past the arytenoid cartilages. The number of intubation attempts was recorded and horses that had a difficult intubation needing more than a single intubation attempt were excluded from further analysis to reduce the bias of injury by the intubation itself. Breath sounds and capnography confirmed correct placement. Horses with a bodyweight of 400–500 kg were intubated using a 26 mm inner diameter tube and horses with a bodyweight of 500–600 kg were intubated using a 30 mm inner diameter endotracheal tube. The endotracheal tube cuff was then inflated via the pilot balloon until a pressure of 40 cmH_2_O was confirmed via manometer. Pulse oximetry, capnography and direct blood pressure measurements were continuously monitored. Airway pressures were maintained within safe clinical limits, and the 40 cmH_2_O endotracheal tube cuff pressure was maintained to provide effective ventilation and minimise pressure on the tracheal wall.

The horses were then positioned on a surgery table in lateral or dorsal recumbency, and a pressure‐limited and pressure‐cycled large animal ventilator (Vet.‐Tec. Model JAVC 2000 J.D. Medical Distributing Company Phoenix, USA) was used for mechanical ventilation. Tidal volume and respiratory rate were standardised to reduce inter‐patient variability. The peak inspiratory pressure (PIP) was maintained between 25 and 30 cmH_2_O, and the frequency was adjusted to maintain the expiratory CO_2_ concentration between 35 and 45 mmHg. At the end of anaesthesia, the vaporiser setting was turned to zero, and mechanical ventilation was stopped. Horses were ventilated by squeezing the re‐breathing bag every 30 s until spontaneous breathing commenced. The horses were disconnected from the circuit and placed into a padded recovery stall in lateral recumbency. Once swallowing, the endotracheal tube cuff was deflated, and the endotracheal tube was removed. Oxygen was insufflated through the nostrils with a flow rate of 15 L min^−1^ and horses were sedated with 0.1–0.2 mg/kg xylazine and assisted during recovery using head‐and‐tail rope support.

Complete physical exams including monitoring of respiratory rate and pattern as well as coughing and nasal discharge were performed every 6 h for the first 3 days post anaesthesia and then every 12 h until discharge from the hospital.

### 2.4 Endoscopic evaluation

Tracheal evaluations were performed using flexible endoscopy prior to general anaesthesia (0) as well as immediately after recovery (R) and 1, 3, 5 and 7 days after general anaesthesia. Lesion severity was scored on a scale from 0 (no lesions) to 4 (severe lesions). The segments assessed included a 15 cm region proximal to the carina and a 25 cm region distal to the larynx.

### 2.5 Data analysis

Due to the limited information in the literature, no formal sample size calculation was performed, but a convenience sample size of 40 animals was chosen. Data were analysed using the statistical software SAS 9.3 (SAS Institute Inc.) and GraphPad Prism Version 7 (GraphPad Software Inc.).

Fleiss' Kappa was used to measure agreement between the three observers, correcting for agreement occurring by chance. As inter‐observer agreement showed excellent agreement with a Fleiss Kappa of 0.88, the mean score values for all the observers were used for further analysis.

Changes of the trachea scores over the five time points were analysed with Friedman's test. Correlation between the degree of trachea irritation at time point 1 (day post general anaesthesia) and duration of intubation, age and weight of the horse was analysed using Pearson correlation. Correlation between the degree of trachea irritation at time point 1 and recumbency during general anaesthesia and medical treatment with antimicrobials and NSAIDs was analysed using Point–Biserial correlation. The level of significance was set at 5% (*p* < 0.05).

## 3 RESULTS

The mean age of enrolled animals was 9.5 ± 6 years and mean bodyweight was 495 ± 78 kg. Eleven horses were positioned in dorsal recumbency and 29 horses in lateral recumbency. The duration of intubation ranged from 60 to 275 min. All horses tolerated the videoendoscopy well. No signs of respiratory disorders and tracheal lesions were observed at time point 0 (prior to intubation).

**FIGURE 1:**
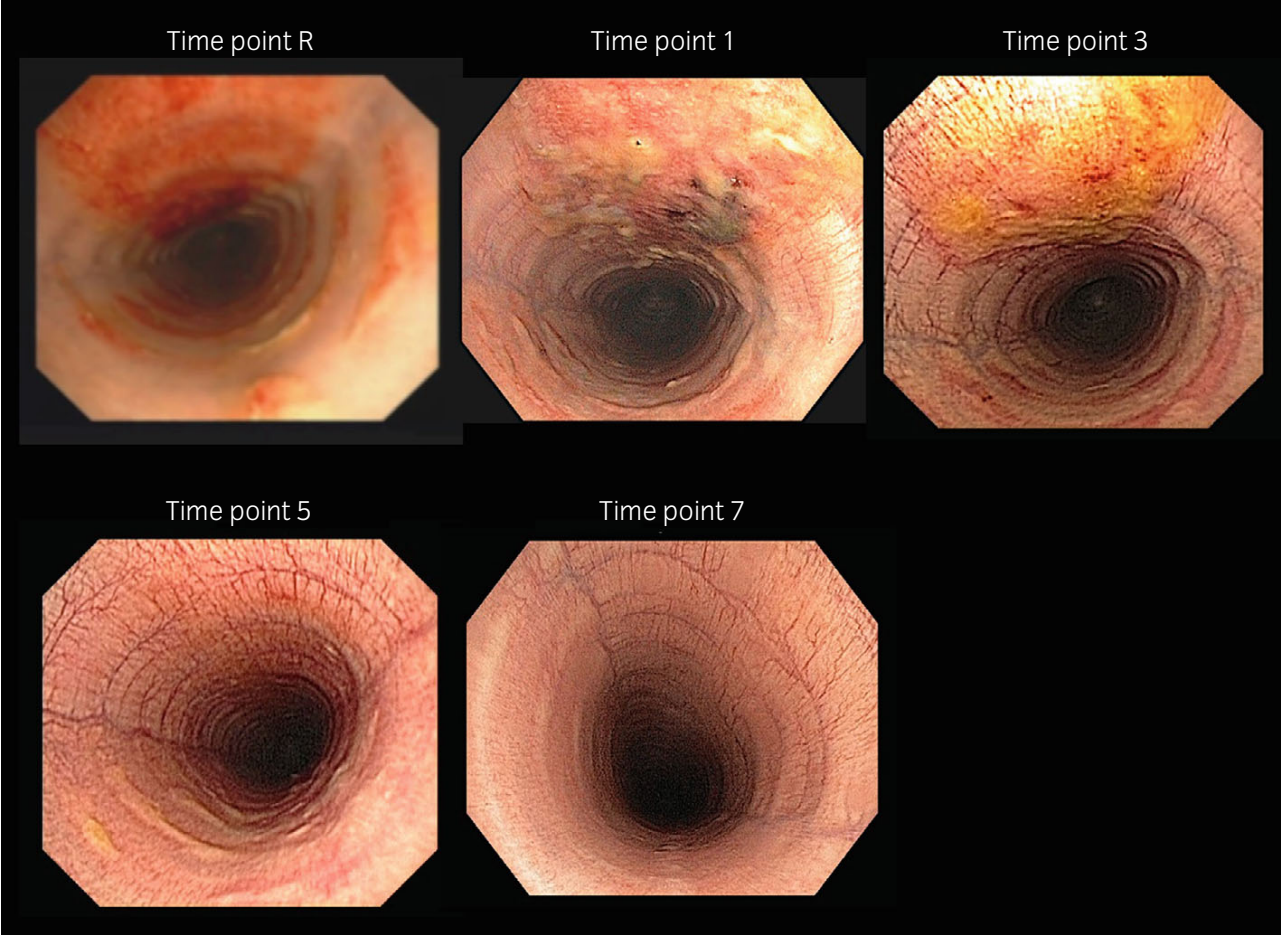
Examples of the degree of tracheal lesions at different time points from one horse enrolled in this study. Timepoints: R = immediately after recovery, 1 = 1 day after general anaesthesia, 3 = 3 days after general anaesthesia, 5 = 5 days after general anaesthesia, 7 = 7 days after general anaesthesia.

**FIGURE 2:**
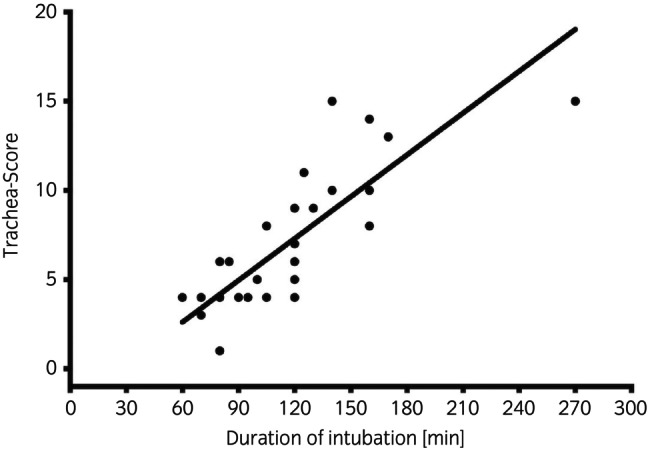
Pearson correlation between degree of tracheal damage at time point 1 and duration of intubation (*r* ^2^ = 0.67, *p* < 0.001) in 40 horses intubated for general anaesthesia.

The endoscopic evaluations revealed varying lesion severities across the sample, with the highest scores typically observed in the dorsal recumbency group. The median (range) score immediately after recovery from anaesthesia (time point R) was 6 (2–12) and increased significantly to 11 (4–15) at time point 1 (*p* = 0.02). The highest degree of trauma at time point 1 was scored as 15. These scores then decreased significantly over time (Table 2). At time point 7, the median score was 0 (0–2) with only four horses showing endoscopic signs of mild tracheitis. Examples of tracheal lesions are shown in Figure 1. It should be noted that all ischaemic lesions were found mid‐trachea and the affected region closely correlated with the position of the endotracheal tube cuff. Duration of intubation correlated positively with the degree of tracheal damage (*r* ^2^ = 0.67, *p* < 0.001) as well as the age of horses (*r* ^2^ = 0.46, *p* < 0.001) for time point 1 (Figure 2). No other parameter showed signs of correlation to severity or duration of tracheal damage.

**TABLE 2:** Median and range of scores indicating the degree of tracheal irritation in 40 horses intubated for general anaesthesia over time.

Time point	Median score	Range	*p* value
0	0	0–0	
R	6	2–12	
1	11	4–15	0.02
3	5	1–11	0.4
5	2	0–5	0.05
7	0	0–2	0.001

*Note*: Timepoints: 0 = prior to intubation, R = immediately after recovery, 1 = 1 day after general anaesthesia, 3 = 3 days after general anaesthesia, 5 = 5 days after general anaesthesia, 7 = 7 days after general anaesthesia. *p* value indicates statistical difference to timepoint R.

Twenty‐two horses received trimethoprim‐sulfadimethoxine 20 mg/kg orally every 12 h perioperatively. Nineteen of these horses were treated for 3 days, and three horses were treated for more than 7 days. All animals received flunixin‐meglumine 1.1 mg/kg intravenously prior to the induction of general anaesthesia. Four horses received one further dose of flunixin‐meglumine 10 h after recovery, 31 horses received flunixin‐meglumine twice daily for 2 more days after general anaesthesia, and 5 horses received flunixin‐meglumine twice daily for 4 more days. There was no significant correlation between the severity of the tracheal lesion at timepoint 1 (day 1 post intubation) and the duration of antimicrobial or antiphlogistic treatment.

## DISCUSSION

All horses recovered well from anaesthesia and no life‐threatening complications of orotracheal intubation were observed in the equine patients included in this study. The ischaemic lesions resulting from intubation peaked in severity approximately 1 day post‐intubation and all resolved within a week. Furthermore, the most strongly associated variables with more severe tracheal wall damage in this study were found to be intubation duration and age of the horse.

In addition to confirming the ubiquitous nature of intubation‐related ischaemic lesions seen in non‐equine studies,^15^ this investigation identified several factors increasing the severity and recovery time of these lesions. All lesions were found approximately mid‐trachea between 25 and 35 cm of the total tracheal length (measured from the larynx), and the length of the affected segment correlated closely with the approximated position and length of the endotracheal tube cuff. This indicates that the cause of this pathology is most likely due to occlusion of the tracheal wall capillary blood flow by the endotracheal cuff. The endotracheal tube cuff was inflated to a pressure of 40 cmH_2_O. This pressure has been described as sufficient to prevent leakage of ventilation gas along the trachea wall.^21^ Cuff pressure is a well‐recognised predisposing factor to tracheal inflammation in human^19^ and veterinary^22^ anaesthesia. A study performed on 10 horses showed significantly more multifocal epithelial lesions and erosions when higher pressures were used.^22^ These cuff pressures were substantially higher (80 and 120 cmH_2_O) compared with the cuff pressures used in the current study. However, the cuff pressure in this study was neither rechecked during general anaesthesia nor adjusted. Further, while cuff pressure can be easily measured, depending on the endotracheal tube material compliance and cuff type, the cuff pressure does not always accurately reflect the pressure exerted by the cuff against the tracheal mucosa; positions of head and neck may influence local pressure peaks.^22^, ^23^ Particularly high‐pressure, low‐volume endotracheal tube cuffs can cause more mucosal irritation and damage compared with low‐pressure, high‐volume tube cuff.^24^ In addition, a study looking at the effects of two different sizes of silicone endotracheal tubes on tracheal and laryngeal mucosa found higher cuff pressures were associated with higher incidents of tracheal circumferential erythema.^25^ In another study, horses intubated with larger (30 mm) tubes showed higher mean scores compared with those with 26 mm tubes, in line with the previous literature that describes increased injury rates with larger tubes.^26^ However, in the present study, the size of the endotracheal tube was not associated with a higher or lower prevalence of tracheal inflammation. As a 28 mm tube was not available in the hospital at the time of the study, a more differentiated impact of tracheal tube size could not be evaluated.

One main factor correlating to a higher lesion severity score was the duration of intubation. In human anaesthesia, post intubation tracheal inflammation and stenosis are often associated with extended periods of intubation.^27^, ^28^ The mainly postulated causative factor is loss of regional blood flow due to cuff pressure on the tracheal wall.^27^ This ischaemic injury begins within the first hour of intubation and progresses with the duration of intubation and cuff pressure exposure.^28^ It might be recommended to reduce the tube cuff pressure at regular time intervals and modify the positioning of the cuff if prolonged intubation is anticipated.

Signs of inflammation at the lesion site peaked at approximately 24 h post‐intubation, and all patients recovered in less than 7 days, which is relatively fast compared with, for example, lesions of the skin.^29^ Patients did not display clinical signs of tracheal lesions or airway pathologies, and mucosal lesions resolved spontaneously, indicating that these were not clinically relevant. In humans, a complication arising from intubation‐associated tracheitis is tracheal stenosis formation.^30^ Tracheal stenosis most commonly results from secondary wound healing with excess granulation and scar tissue formation around the tracheal lumen site developing within 3–6 weeks after intubation.^28^ Therefore, if in horses the lesions do not resolve within the expected time, this could be indicative of significant trauma or infection that can potentially result in more severe complications like mucosal necrosis and scar tissue formation that results in stenosis if not treated accordingly.^31^, ^32^, ^33^

Although this study identified factors that could potentially impact the severity of intubation‐related tracheal ischaemic lesions, several limitations should be considered as they may have impacted the investigation's outcome. Some potentially confounding factors, such as intraoperative PaO_2_ and mean arterial blood pressure, were not analysed. Further, other confounding factors might exist that this study was not able to identify. Ideally, multicentre studies with larger numbers of enrolled patients are necessary to evaluate the effects of these and other confounding factors on the prevalence of tracheal lesion formation after intubation for general anaesthesia. Further, a Cook silicone endotracheal tube was used for all patients, and tubes were cleaned variably between uses. Potential differences in cleaning methods and residues of disinfectant could have led to variability in the level of inflammation observed in the trachea. Although not observed, mechanical irritation by the videoendoscope could also have potentially augmented the degree of observed inflammation. Additionally, all animals received NSAIDs for a variable time, and 50% of the horses received antimicrobials, which could have impacted the appearance of the ischaemic lesions. In our study, neither the administration of antimicrobials nor the duration of NSAID administration had an impact on severity nor duration of healing in these horses. However, given the relatively small sample size and lack of power analysis, the authors cannot exclude a potential effect of these treatments and lesion healing. A larger clinical trial with more regulated treatment is necessary to fully explore the impact of antimicrobial and anti‐inflammatory treatment on tracheal lesion healing. Endotracheal tube cuff pressure has also been shown to have an impact on the degree of trauma in mammals.^3^, ^4^, ^5^, ^6^ Although the cuff pressures were 40 cmH_2_O after anaesthesia induction, the cuff pressure was not continuously monitored, so variations in pressure may have been overlooked. The use of high‐volume, low‐pressure cuffs has been standardised to reduce cuff‐pressure‐related tracheal injury, but further improvement of the cuff shape could help minimise the possible damage to tracheal mucosa. One such modification is the use of a tapered cuff shape, instead of the conventionally used cylindrical endotracheal tube cuff, which has been shown to reduce pressure‐related trauma to the tracheal mucosa.^19^, ^34^, ^35^ Due to the non‐fully circular cross‐section of the trachea in the relevant region, an oval to elliptic cross‐section of the inflated cuff would be a valid option to distribute excentric forces more evenly on the mucosal surface.^16^, ^17^ In this context, the vascularisation pattern of the tracheal lining also plays a potential role regarding lesion distribution. Further, a clinical report in a horse undergoing myelography indicated that the movement of the endotracheal tube can be associated with tracheal mucosal damage.^36^ While in the study the endotracheal tubes were not moved or repositioned after the horses were placed on the surgery table, horses had to be moved onto the table after intubation, and therefore this contributing factor cannot be fully excluded.

This study suggests that subclinical tracheal ischaemic lesions related to orotracheal intubation are common in horses and appear to be positively associated with the duration of intubation. This study was not able to identify a safe cutoff time that would indicate a lower risk for intubation‐associated lesions. Although these ischaemic lesions generally do not lead to clinical signs or impact overall patient health, complications could arise in cases where tracheal mucosa trauma is excessive, potentially leading to significant tissue changes during healing. Continued advancements in intubation equipment may help to mitigate trauma to the tracheal mucosa, potentially reducing the risk of complications and improving outcomes for intubated patients.

## FUNDING INFORMATION

This study was funded using intramural support from the University of Veterinary Medicine Hannover, Foundation, Clinic for Horses.

## CONFLICT OF INTEREST STATEMENT

The authors declare no conflicts of interest.

## AUTHOR CONTRIBUTIONS

**Aiden Parente:** Writing – original draft; project administration. **Florian Geburek:** Conceptualization; investigation; methodology; writing – review and editing. **Sabine Kästner:** Conceptualization; investigation; methodology; writing – review and editing. **Charlotte Iversen:** Conceptualization; investigation; methodology; writing – review and editing. **Klaus Hopster:** Conceptualization; investigation; funding acquisition; methodology; writing – review and editing; formal analysis; project administration; data curation; supervision.

## DATA INTEGRITY STATEMENT

Klaus Hopster had full access to all the data in the study and takes responsibility for the integrity of the data and the accuracy of the data analysis.

## ETHICAL ANIMAL RESEARCH

The study protocol was approved by the Ethics Committee for Animal Experiments of Lower Saxony (33.9‐42502‐05‐14A467).

## INFORMED CONSENT

Owners gave consent for their animals' inclusion in the study.

## PEER REVIEW

The peer review history for this article is available at https://www.webofscience.com/api/gateway/wos/peer-review/10.1111/evj.14487.

## Data Availability

The data that support the findings of this study are openly available in DRYAD (https://doi.org/10.5061/dryad.3n5tb2rv6), reference number 5061.
